# Cationic Polymer Nanoparticles-Mediated Delivery of miR-124 Impairs Tumorigenicity of Prostate Cancer Cells

**DOI:** 10.3390/ijms21030869

**Published:** 2020-01-29

**Authors:** Raffaele Conte, Anna Valentino, Francesca Di Cristo, Gianfranco Peluso, Pierfrancesco Cerruti, Anna Di Salle, Anna Calarco

**Affiliations:** 1 Research Institute on Terrestrial Ecosystems (IRET)—CNR, Via Pietro Castellino 111, 80131 Naples, Italy; raffaele.conte86@tiscali.it (R.C.); gianfranco.peluso@cnr.it (G.P.); anna.disalle@cnr.it (A.D.S.); 2 Elleva Pharma s.r.l. via P. Castellino, 111 – 80131 Naples, Italy; anna.valentino@ellevapharma.com (A.V.); francesca.dicristo@ellevapharma.com (F.D.C.); 3 Institute for Polymers, Composites and Biomaterials (IPCB-CNR) Via Campi Flegrei, 34, 80078 Pozzuoli (NA), Italy

**Keywords:** polymer nanoparticles, aminolysis, miR-124, gene delivery, polyhydroxybutyrate (PHB), polyethyleneimine (PEI), prostate cancer

## Abstract

MicroRNAs (miRNAs) play a pivotal role in regulating the expression of genes involved in tumor development, invasion, and metastasis. In particular, microRNA-124 (miR-124) modulates the expression of carnitine palmitoyltransferase 1A (CPT1A) at the post-transcriptional level, impairing the ability of androgen-independent prostate cancer (PC3) cells to completely metabolize lipid substrates. However, the clinical translation of miRNAs requires the development of effective and safe delivery systems able to protect nucleic acids from degradation. Herein, biodegradable polyethyleneimine-functionalized polyhydroxybutyrate nanoparticles (PHB-PEI NPs) were prepared by aminolysis and used as cationic non-viral vectors to complex and deliver miR-124 in PC3 cells. Notably, the PHB-PEI NPs/miRNA complex effectively protected miR-124 from RNAse degradation, resulting in a 30% increase in delivery efficiency in PC3 cells compared to a commercial transfection agent (Lipofectamine RNAiMAX). Furthermore, the NPs-delivered miR-124 successfully impaired hallmarks of tumorigenicity, such as cell proliferation, motility, and colony formation, through CPT1A modulation. These results demonstrate that the use of PHB-PEI NPs represents a suitable and convenient strategy to develop novel nanomaterials with excellent biocompatibility and high transfection efficiency for cancer therapy.

## 1. Introduction

Prostate cancer (PC) remains a significant leading cause of male death in developed occidental countries [1,2]. Current PC therapies (e.g., surgery, hormonal therapy, radiation, or chemotherapy, etc.) aim to prolong patient survival, improving and maintaining quality of life. However, more than 80% of PC patients develop resistance to androgen deprivation therapy (ADT), a status named castration-resistant PC, leading to disease progression and metastases with poor survival [3].

Recently, the pivotal role of microRNAs (miRNAs) in PC initiation and progression as well as in modulating the response of PC to treatments has been underlined [4,5,6], suggesting their use as diagnostic biomarkers and alternative treatments [5,7,8,9,10]. In particular, microRNA-124 (miR-124) is a class of small non-coding RNAs significantly implicated in several types of human cancers, including hepatocellular carcinoma, glioblastoma, osteosarcoma, and breast cancer [11,12,13].

Several studies reported the suppressive role of miR-124 on prostate cancer cell proliferation, apoptosis, and metastasis [14,15,16]. Shi et al. showed that low expression of miR-124 contributes to the pathogenesis of PC via increased expression of the androgen receptor [17], while Kang et al. demonstrated miR-124 anti-proliferative and anti-aggressive effects related to the inhibition of PACE4 transcription [18]. Our previous study provided evidence that miR-124 is involved in PC deregulation of mitochondrial fatty acid (FA) oxidation via carnitine system modulation, targeting carnitine-palmitoyltransferase-1 A (CPT1A) [18]. Forced expression of the synthetic miR-124 mimic in androgen-dependent and androgen-independent PC cells (LNCaP and PC3, respectively) affects tumorigenic properties, regardless of their hormone sensitivity. Together, these studies suggest that administration of an miR-124 mimic may represent a promising therapeutic tool for PC management. However, the successful translation of miRNA therapy from bench to bedside is still a severe challenge due to the short half-life and poor stability of miRNA mimics. Indeed, their clinical translation requires effective delivery systems able to enhance cellular uptake in the tumor site as well as able to protect nucleic acids from degradation.

Nanoparticle (NP)-based targeted delivery, such as the use of cationic polymeric nanoparticles, represents the most promising solution to protect miRNAs from endosomal and/or lysosomal degradation, thus achieving their therapeutic effects [19,20,21,22]. Recently, few studies reported the therapeutic potential of miR-124 polymeric nanoparticles (NPs) in neurodegenerative disorders. Saraiva et al. fabricated polylactic-co-glycolic acid (PLGA) NPs containing perfluoro-1,5-crown ether and coated with protamine sulfate to complex microRNA-124. This formulation was able to enhance brain repair in in vitro and in vivo model of Parkinson’s disease, leading to the amelioration of motor symptoms in mice treated with 6-hydroxydopamine [23]. In addition, Louw et al. synthetized chitosan miRNA-124 polyplexes that reduced neuronal inflammation in rat models of spinal cord injury [24]. To our knowledge, only Shi et al. reported the delivery of miR-124 in prostate cancer, as JetPEI (linear PEI derivative) complexes. The authors demonstrated that the intravenous administration of miR-124 polyplex inhibited the growth of androgen-dependent and -independent prostate cancer cells and increased tumor cell apoptosis in an enzalutamide-resistant xenograft model [16]. However, the clinical translation of JetPEI as a delivery vehicle presents a major hurdle, requiring appropriate drug formulation and optimization.

Several natural and synthetic biodegradable polymers have been employed in the synthesis of micro- and nano-vectors for nucleic acids delivery. For this purpose, mainly polysaccharides (such as dextran, cyclodextrins, chitosan), polyhydric polymers (polyvinyl alcohol, polyethylene glycol), and polyesters (PLGA, polycaprolactone, polylactic acid) have been utilized [25,26,27].

Poly(3-hydroxybutyrate) (PHB) has emerged as an alternative to the above mentioned biodegradable polymers because of its unique physiochemical and mechanical properties [28,29]. PHB exhibits excellent biocompatibility and hemocompatibility, generating a mild foreign-body response with no activation of the complement system. However, although the hydrolytic and enzymatic (via nonspecific lipases and esterases) in vivo degradation processes of PHB make this polymer a good candidate as an efficient delivery system [30,31], its application in gene delivery is limited due to its negative surface charge. Polyethyleneimine (PEI), a water-soluble polymer with protonable amino groups, has been widely used for nucleic acids complexation through electrostatic interactions [32,33,34]. However, its high transfection efficiency is frequently related to cellular toxicity due to polymer interference with cellular nuclear processes [35].

The present study reported, for the first time, the synthesis of PHB nanoparticles (PHB NPs), functionalized with low molecular weight PEI via aminolysis, and their use as effective cationic non-viral vectors to complex and deliver miR-124 in prostate cancer cells. The wet-chemical modification of polymeric surface prevented free PEI from being released in the living cell after delivery of the nucleic acid. Furthermore, the functionalization of the polymer via aminolysis represents a simple, rapid, and environmentally-safe procedure, which is easily scalable to an industrial environment. The physicochemical properties of cationic nanoparticles, such as the particle size, zeta potential, morphology, and stability, were evaluated. Additionally, the biocompatibility, cellular uptake, and delivery efficiency of PHB-PEI NPs/miR-124 complexes were assessed in human prostate cancer cells, PC3, used as an in vitro cell model.

Our results showed that PHB-PEI NPs enhance cellular uptake of miR-124, in comparison to a commercially available Lipofectamine gene delivery system, decrease CPT1A expression and inhibit PC3 proliferation, migration, and invasion. Therefore, the high transfection efficiency and low cell toxicity make PHB-PEI NPs a promising delivery system for the treatment of prostate cancer in clinical trials.

## 2. Results and Discussion

### 2.1. Nanoparticles Synthesis and Characterization

Recently, scientific evidence established the master regulator role of miRNAs in tumor initiation, progression, and metastasis, shedding light on miRNAs as a promising strategy for cancer therapy. In comparison to ‘one-drug-one-target’ treatments (i.e., chemotherapeutic and/or radiotherapeutic agents), miRNA-based therapies offer the advantage of concurrently regulating multiple cellular targets, restoring or repressing miRNA expression and activity [10]. However, miRNAs clinical translation results in several off-target effects due to poor miRNA pharmacokinetic profile, rapid in vivo clearance, and limited delivery at the tumor site. Cationic polymeric vectors could potentially overcome these limitations, protecting nucleic acid from enzymatic degradation and increasing their bioavailability and loading efficiency. Therefore, they are regarded as efficient tools in in vitro and in vivo miRNA systemic delivery for cancer therapy [36].

**Scheme 1:**
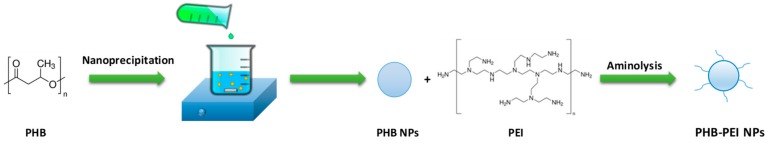
Preparation route of polyethyleneimine-functionalized PHB nanoparticles (PHB-PEI NPs) by nanoprecipitation and aminolysis.

Due to its high cationic charge density at physiological pH, polyethyleneimine (PEI) is a potential candidate for gene delivery. Indeed, it is well known that PEI induces a rapid intracellular uptake and miRNA release via an endocytic mechanism (‘proton sponge effect’) [37]. However, this strong positive surface charge causes severe cytotoxicity and nonspecific binding with serum [38]. To exploit the high transfection efficiency of PEI while limiting cellular toxicity, herein a two-step procedure, including preparation of PHB NPs followed by surface modification with PEI, was developed for the preparation of PHB-PEI NPs (Scheme 1).

**Figure 1:**
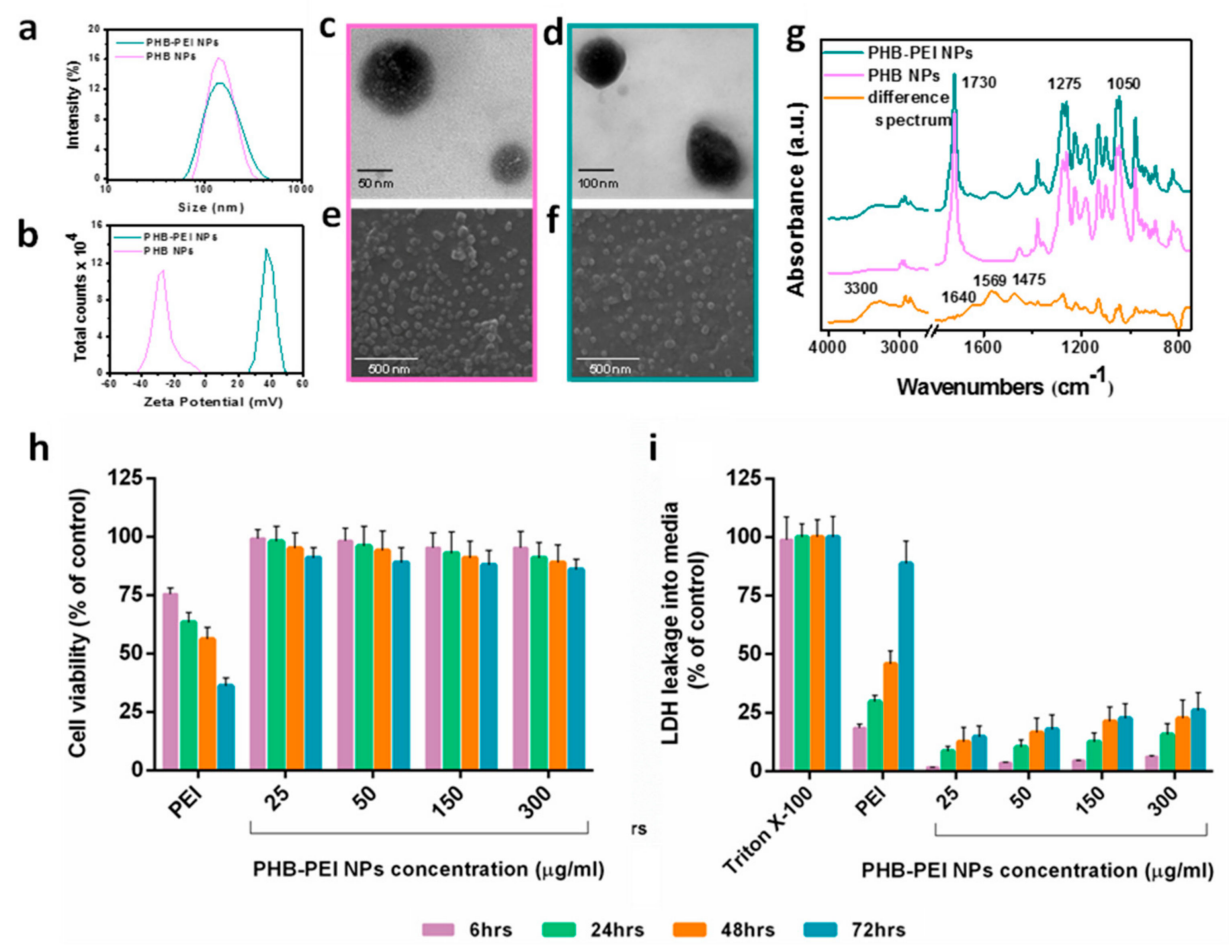
Size, morphological, chemical characterization, and cytotoxicity of PHB based NPs. (**a**) Size distribution and (**b**) zeta potential of PHB and PHB-PEI NPs. TEM and SEM images of: (**c**, **e**) PHB, and (**d**, **f**) PHB-PEI NPs. (**g**) FTIR-ATR spectra of PHB and PHB-PEI NPs along with difference spectra. (**h**, **i**) In vitro cytotoxicity of PHB-PEI NPs. Cytotoxicity was determined in PC3 cells after 6, 24, 48, and 72 h of incubation with varying concentrations of PHB-PEI NPs using: (**h**) the Cell Counting Kit-8 (CCK-8) assay, and (**i**) the Lactate Dehydrogenase (LDH) assay. Untreated cells were used as control. Data are presented as the mean ± SD for three independent measurements.

PHB NPs were prepared in Pluronic F-127 aqueous solution by nanoprecipitation. First, the optimal process parameters were determined to achieve NPs with the desired size and narrow polydispersity index (PDI). As reported in Table S1, the mean particle size ranged from 138 to 705 nm and was strongly affected by polymer and surfactant concentration. In particular, the NPs size was directly and inversely proportional to PHB and Pluronic F-127 concentrations, respectively. Indeed, larger nanoparticles formed at higher polymer concentrations due to lower diffusion coefficient of solvent in water [39] and, when an insufficient amount of surfactant resulted in a reduced interfacial stability, leading to particle coalescence. According to the Dynamic Light Scattering (DLS) results, PHB NPs obtained using 8.33 mg/mL PHB and 2 wt % Pluronic F-127 solutions, which showed a hydrodynamic diameter of 138 nm and moderate dispersity (Figure 1a and Supplementary Table S1), were selected for the subsequent surface modification reaction. Aminolysis is an easy-to-perform chemical modification which allows the engraft of free –NH_2_ groups along the polyester chains [31,40].

Upon aminolysis, one of the amino groups of branched PEI reacts with the ester group of PHB to form an amide bond, leaving the others amine groups of PEI available for nucleic acids complexation. Aminolysis was performed by immersing pristine PHB NPs in PEI–isopropanol solution at varying concentrations (12%, 24%, 48% w/v) for regular intervals, up to a maximum of 60 min ( Supplementary Table S2). The treatment with 24% PEI for 15 min at 50 °C resulted in the highest amount of –NH_2_ groups on the PHB NPs surface (38.2 mmol -NH_2_/g) as determined by quantitative ninhydrin assay. DLS analysis demonstrated a slight increase in hydrodynamic diameter (151.6 ± 62.8 nm) (Figure 1a), while the zeta potential dramatically increased following aminolysis, from −26.8 ± 6.9 to 37.1 ± 2.5 mV (Figure 1b), confirming the successful surface modification.

Size and morphology of the NPs were further characterized by TEM and SEM, which showed that they were spherical and regular in size (around 150 nm) with a smooth surface (Figure 1c,d). Electron microscopy did not show any significant size differences between PHB NPs and PHB-PEI NPs and the PEI coating was not clearly distinguishable (Figure 1e). SEM also confirmed that PHB nanoparticles maintained their low propensity to agglomerate even after surface modification (Figure 1f). Indeed, both PHB NPs and PHB-PEI NPs were easily redispersed in water after isolation.

Chemical characterization of the NPs was performed by FTIR spectroscopy. FTIR-ATR spectra of pristine PHB NPs and PHB-PEI NPs are reported in Figure 1g, along with their difference spectra. The PHB NPs spectrum showed the presence of main absorption peaks of PHB at 2980 and 2940 cm^−1^ due to the aliphatic backbone at 1730 cm^−1^, ascribed to the ester carbonyl stretching, and at 1275 cm^−1^, corresponding to the bending of C-H bonds. Moreover, a pattern of bands between 1000 and 1250 cm^−1^ accounted for the stretching of the C-O bond of the carboxyl groups [41]. PHB-PEI NPs and the difference spectrum clearly revealed absorption peaks that can be assigned to grafted PEI. Specifically, amide I and amide II stretching bands at 1640 and 1568 cm^−1^, respectively, were noticed, along with the peak at 1475 cm^−1^, indicative of the in-plane bending of CH_2_ groups. Moreover, the broad absorption at 3300 cm^−1^ was assigned to the stretching vibration of primary amines [42].

PEI transfection is accompanied with certain degrees of cytotoxicity, depending on polymer molecular weight. PEI may induce cell cytotoxicity due the cell membrane destabilization and/or by interfering with host-gene expression in the nucleus [43]. However, it has been demonstrated that low molecular weight PEI shows lower cytotoxicity compared to high molecular weight PEI [34,44]. In order to determine if aminolysis can reduce the toxicity of PEI, the biocompatibility of PHB-PEI NPs was assessed after incubation of cells with varying concentrations of NPs. CCK-8 and LDH assays were performed on different cell lines (Caco-2, MCF-7, MCF10A, and PC3 cells), for up to 72 h. As reported in Figure 1h and Figure S1a–c, no changes in cell viability were observed in all cell lines after 72 h of incubation (>85% cell viability) with respect to the control. On the contrary, cells treated with PEI (25 μg/mL) showed a significant reduction in cell viability (*p* < 0.01). Moreover, the addition of PHB-PEI NPs to cell cultures induced only minimal or negligible damage to cell membrane integrity, as evidenced by the low LDH release even after 72 h at all tested concentrations (Figure 1i and Supplementary Figure S1d–f). The reported results confirmed that aminolysis considerably reduced the PEI-induced toxicity by preventing the release of free toxic primary amino groups in the cell.

### 2.2. Characterization of PHB-PEI NPs/miR-124 Complexes (miR-124 NPs)

**Figure 2:**
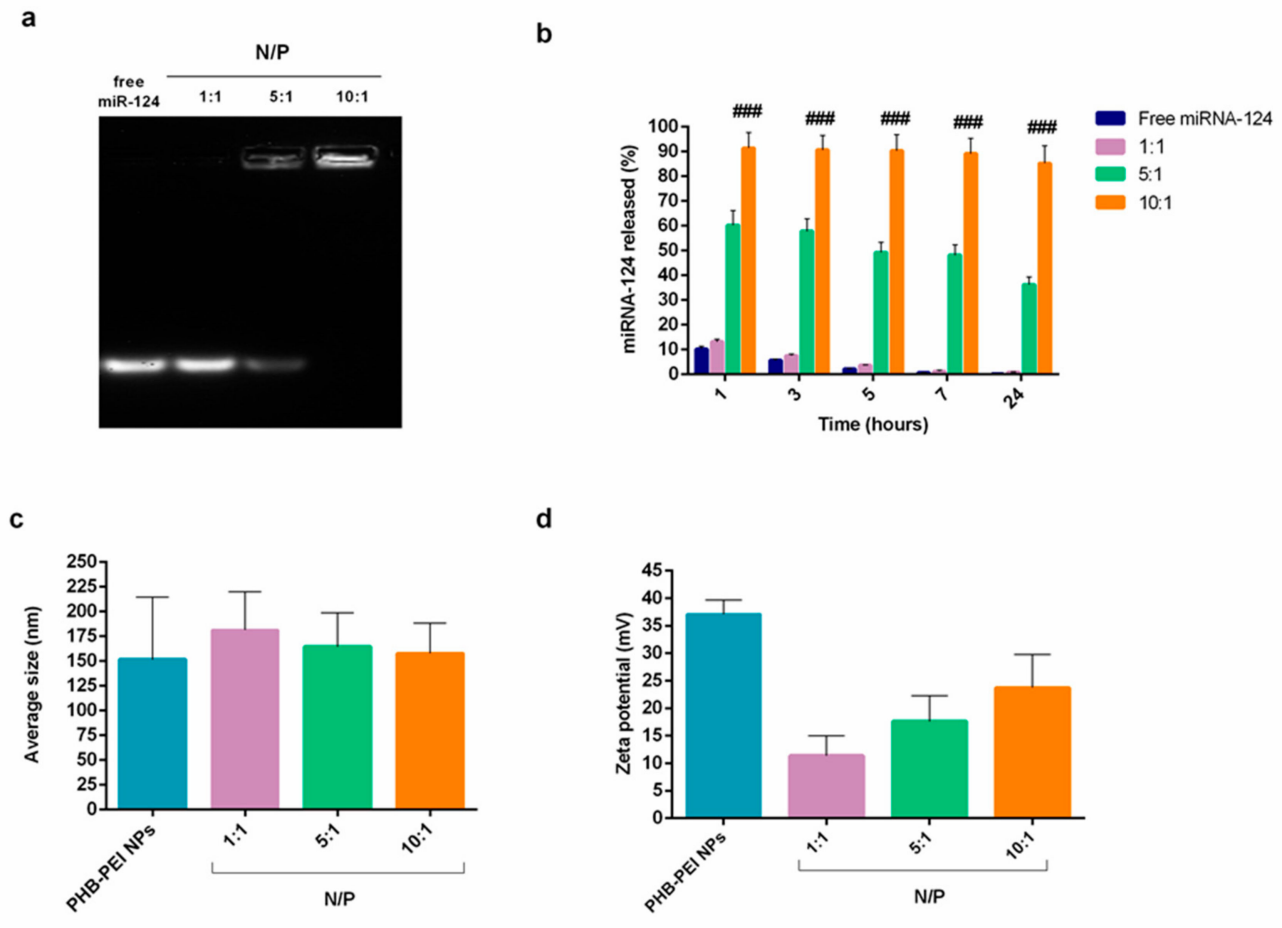
Characterization of PHB-PEI NPs/miR-124 complexes. (**a**) Electrophoretic mobility of miR-124 NPs at different N/P ratios. (**b**) miRNA-124 release profile evaluated by qRT-PCR analysis for 24 h in growth medium. Free miR-124 was used as control. (**c**) Average hydrodynamic diameter and (**d**) zeta potential of miR-124 NPs at different N/P ratios (mean ± SD, *n* = 6). Statistically significant variations: ### *p* < 0.001 N/P 10:1 versus N/P 5:1, N/P 1:1, and free miR-124.

The ability of PHB-PEI NPs to electrostatically interact, at different N/P ratios (1:1, 5:1, 10:1), with phosphate groups on the miRNA backbone was determined by gel retardation assay. As depicted in Figure 2a, the intensity of migrating free miRNA decreased gradually with an increase in the N/P ratio. In particular, PHB-PEI NPs were able to condense miRNA already at an N/P ratio of 5, forming a stable miRNA/NPs complex at an N/P of 10.

As a prerequisite to obtaining an efficient miRNA delivery system for therapeutic applications, the cationic NPs should protect nucleic acids from nuclease degradation both in serum and extracellular matrix [21]. q-PCR data (Figure 2b) revealed that ~90% of intact miR-124 was also detected after 24 h of incubation in the growth medium when complexed with PHB-PEI NPs at an N/P ratio of 10, while free miR-124, used as control, was already completely degraded after 1 h of incubation (Supplementary Figure S2). These results demonstrated that PHB-PEI NPs are able to protect nucleic acid from nuclease degradation for extended period of times.

The cellular internalization of the complex is modulated by its physico-chemical properties, such as particle size and zeta potential [45,46,47]. Therefore, the nanocomplexes’ average hydrodynamic diameter and zeta potential were determined by dynamic and electrophoretic light scattering, respectively. As shown in Figure 2c, at a low N/P ratio, particles larger than pristine PHB-PEI NPs formed (hydrodynamic diameter = 181.4 ± 38.6 at N/P = 1), whose size tended to decrease when the N/P ratio increased, reaching the value of 157.6 ± 30.8 at N/P = 10. In addition, at N/P ratio = 1, the strongly positive zeta potential of the NPs complex declined (10.47 ± 1.27 mV) due to the presence of negatively charged miRNA on NPs surface (Figure 2d). However, at N/P = 10, the surface charge of the nanocomplex increased considerably, confirming the ability of PHB-PEI NPs to completely complex miRNA, in accordance with the gel retardation results.

### 2.3. Cellular Uptake of miR-124 NPs

**Figure 3:**
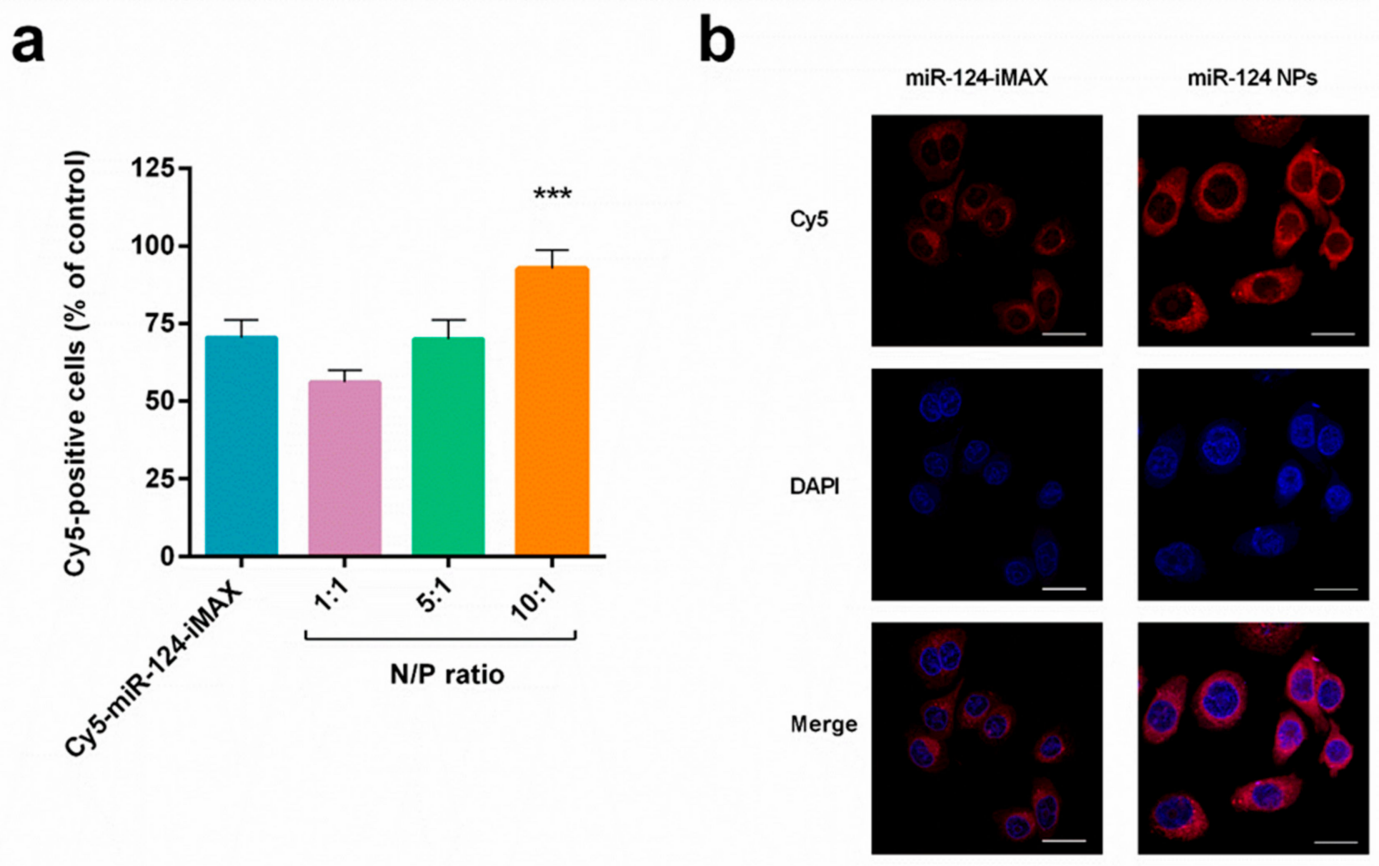
Intracellular delivery efficiency of PHB-PEI NPs/miR-124 complexes. (**a**) PC3 uptake of miR-124 NPs analyzed by Fluorescent Activated Cell Sorting (FACS). To facilitate observations, miR-124 was labeled with the Cy5 fluorescent probe (red) to form Cy5-miR-124-NPs at different N/P ratios (1:1 to 10:1). Lipofectamine RNAiMAX (iMAX) was used as a control. Cells were incubated with fluorescent nanocomplexes for 4 h, washed with PBS, and incubated for another 20 h prior to analysis by flow cytometer (mean ± SD, *n* = 6). Statistically significant variations: *** *p* < 0.001 N/P 10:1 versus N/P 5:1, N/P 1:1, and iMAX. (**b**) Fluorescence images of PC3 cells incubated with Cy5-miR-124-NPs (N/P ratio 10:1) or Cy5-miR-124-iMAX for 4 h. Magnification 40×, scale bar is 50 μm.

Several reports have shown that miRNAs can be efficiently delivered into the cancer cell by nano-sized, non-viral vectors, minimizing the poor cellular uptake of free nucleic acids due to the charge repulsion between the cell membrane and miRNAs [48]. Inter alia, Shi et al. reported the delivery of miR-124 in prostate cancer as JetPEI complexes. The authors demonstrated that the intravenous administration of miR-124 polyplex inhibited the growth of androgen-dependent and -independent prostate cancer cells and increased tumor cell apoptosis in an enzalutamide-resistant xenograft model [16]. However, the clinical translation of JetPEI as a delivery vehicle requires appropriate drug formulation and optimization to avoid cytotoxic effects. To investigate the role of PHB-PEI NPs in miR-124 intracellular delivery, the transfection efficiency of miR-124 NPs was assessed by flow cytometry. Lipofectamine RNAiMAX (iMAX) was used as a control. Interestingly, in comparison with miRNA transfected using the commercial transfection agent (Cy5-miR-124-iMAX), Cy5-miR-124 NPs at an N/P ratio 10:1 induced a significant (*p* < 0.01) 30% increase in Cy5-positive PC3 (92.6 ± 6.20% and 70.5 ± 5.63%, respectively, Figure 3a). Furthermore, the transfection at N/P ratios below 10 resulted in a low signal inside cells, probably due to the weak interactions between NPs and miRNA. Fluorescence microscopy was also performed to evaluate subcellular distributions of Cy5-miR-124 in PC3 cells. As depicted in Figure 3b, a strong and diffuse fluorescence was observed in the cytoplasm of cells treated with the nanocomplex compared to PC3 incubated with lipofectamine. These results confirmed the advantages of using nontoxic PHB-PEI NPs to remarkably enhance miRNA delivery.

### 2.4. Anti-Cancer Effects of miR-124 NPs

Lipid metabolism dysregulation, due to abnormal expression of various genes, proteins, and signaling pathways, plays a pivotal role both in carcinogenesis and some metabolic syndromes [49,50,51]. Prostate cancer cells differ from many other cancer types in that they promote fatty acid oxidation (FAO) to fuel their energy and metabolic intermediate needs, even under nutrient-replete conditions. Indeed, mitochondrial FAO produces much more ATP/mole than oxidation of glucose or amino acids [49]. Several studies reported that carnitine palmitoyltransferase 1A (CPT1A), the key enzyme of mitochondrial FAO in both healthy and cancer cells, is strongly expressed in several cancers, including the hormone-dependent breast and prostate cancers [52]. CPT1A transfers the acyl group of a long-chain fatty acyl-CoA from coenzyme A to carnitine. Evidences have shown that the downregulation of CPT1A via inhibition/depletion impairs cancer cell proliferation in lung, gastric, prostate, lymphoma, and leukemia [53,54,55]. Schlaepfer et al. demonstrated that etomoxir, an irreversible inhibitor of CPT1A, is able to induce cytotoxicity in the androgen-dependent prostate cell lines as well as patient-derived prostate cancer cells [56,57]. Moreover, pharmacological blockage of CPT1A leads to a dose- and time-dependent cell growth reduction and apoptosis in leukemia cell lines and primary hematopoietic malignant cells [58].

**Figure 4:**
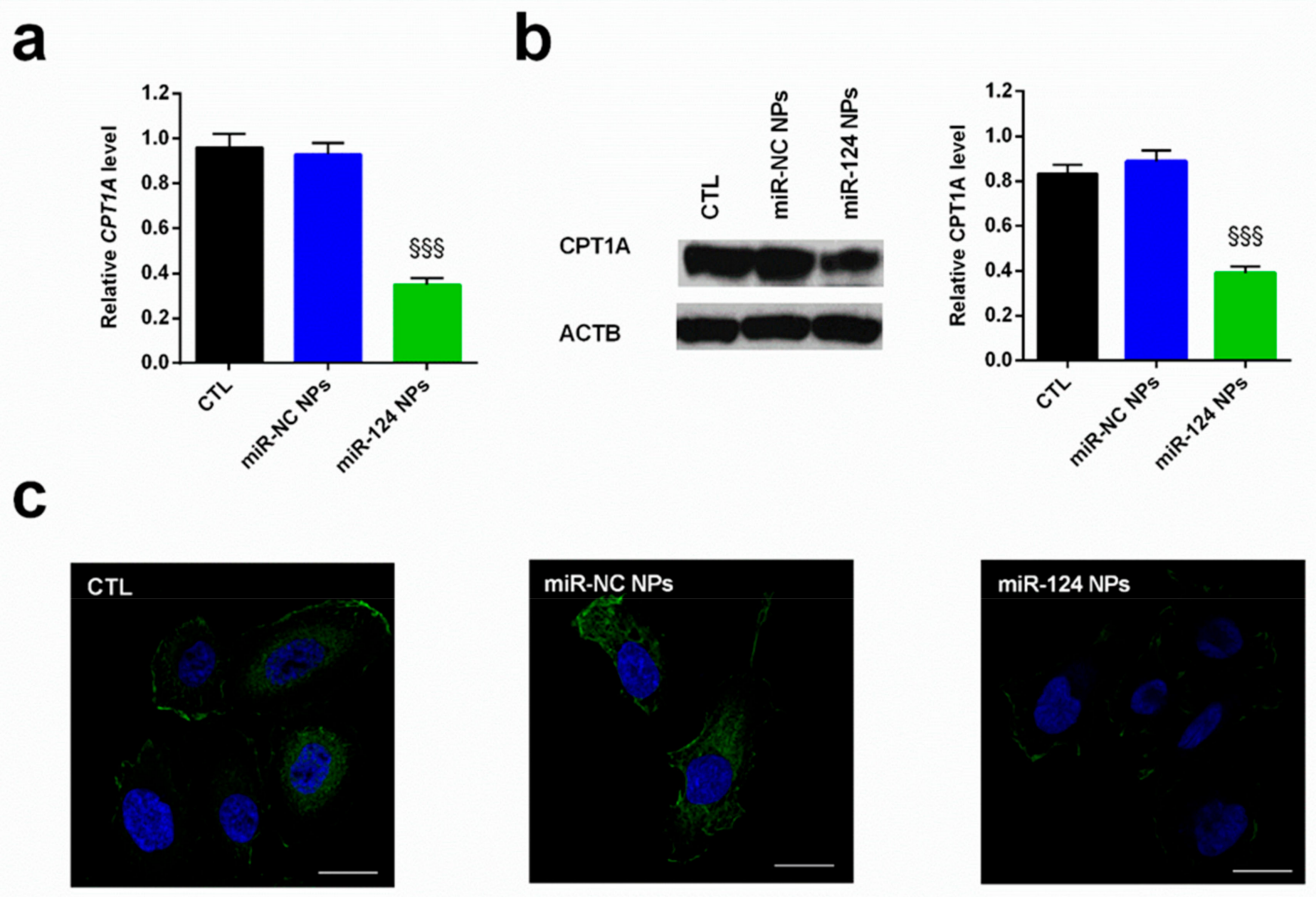
Expression of CPT1A in the PC3 cells in the presence of nanocomplexes. Cells were cultured for 24 h after transfection with miR-124-NPs or miR-NC-NPs at a 10:1 N/P ratio. The untreated cells were used as control (CTL). (**a**) miRNA target level (CPT1A) was quantified by quantitative real-time PCR (qPCR) and normalized to actin (ACTB) as a housekeeping gene. The comparative cycle threshold (CT) method (2^−∆∆*C*t^) was applied to calculate relative differences in qPCR results. (**b**) Western blot analysis of CPT1A protein was performed on total PC3 protein fraction. The protein expression was normalized to the housekeeping protein ACTB. The bars represent the mean ± SD, *n* = 3. Statistically significant variations: §§§ *p* < 0.001 miRNA versus miR-NC and CTL. (**c**) Representative fluorescent images of CPT1A expression. Immunofluorescence stain was performed with a FITC-labeled secondary antibody and counterstained with DAPI. Magnification 40×, scale bar is 50 μm.

Our previous research revealed that in prostate cancer cells, such as PC3, the downregulation of miR-124 induces an increase in both the expression and activity of CPT1A, allowing malignant cells to be more prone to fatty acid utilization with respect to normal cells [29]. Altogether, these observations suggest that lipid oxidation is an important player in the plasticity of cancer cells, allowing them to survive even in harsh environments such as androgen deprivation [59,60]. Based on the results reported above, miR-124 NPs at N/P = 10 was employed in the study. The functionality of the miR-124 released by PHB-PEI NPs was determined by evaluating the expression level of CPT1A by both qPCR (Figure 4a) and Western blot (Figure 4b) analyses. As reported in Figure 4, a significant (*p* < 0.001) downregulation of the investigated target was observed 24 h after transfection at both transcriptional and translation level compared to non-transfected cells (CTL) or miR-NC-NPs-mediated transfection. In addition, the CPT1A immunofluorescence stain revealed a low expression in cells treated with miR-124 NPs with respect to control cells (Figure 4c).

**Figure 5:**
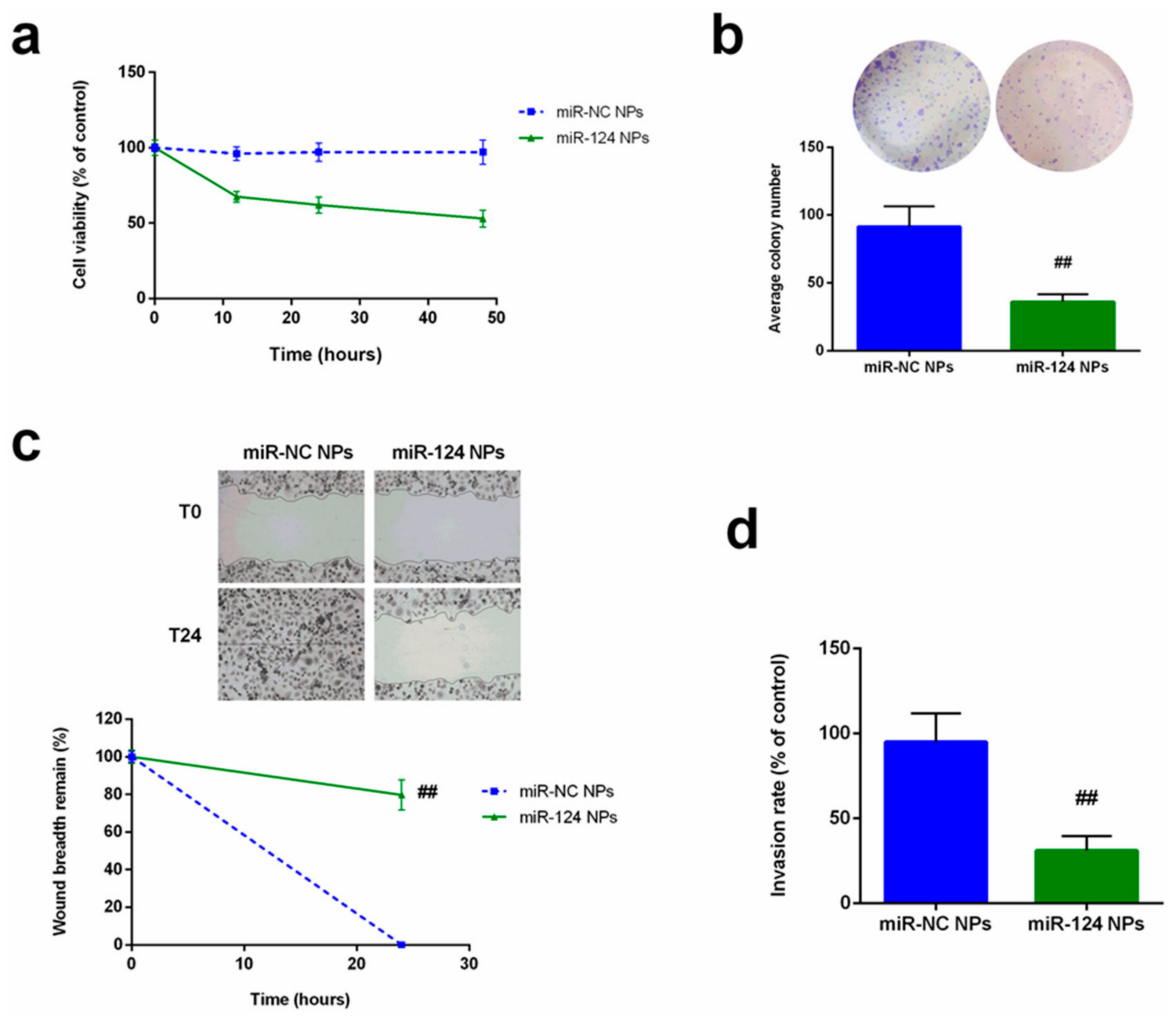
Influence of forced expression of miR-124 on PC3 proliferation, migration, and invasion. (**a**) Cell proliferation of PC3 cells was determined by CCK8 assay after 12, 24, and 48 h. (**b**) Colony formation assay was performed after 14 days of culture. For quantification, colonies with at least 50 cells were considered. Representative micrographs were obtained using phase contrast microscope after staining with crystal violet. (**c**) Wound-healing assay performed on transfected PC3 cell, and wound closure rate measured by detecting the closure distance after 24 h. Representative micrographs of the cell migration (top) and quantification (bottom) from three independent experiments are presented. (**d**) Transwell invasion assay with Matrigel performed in miR-124-NPs or miR-NC-NPs-transfected PC3 cells after 24 h. Five random fields in each well were counted under a microscope. The bars represent the mean ± SD, *n* = 3. Statistically significant variations: ## *p* < 0.01, miRNA versus miR-NC.

The ability of miR-124 NPs to interfere with malignant cell functionality (cell proliferation, motility, and colony formation) was confirmed on PC3 cells transfected with miR-124 NPs as well as miR-NC (Figure 5). As reported in Figure 5a, the cell growth rate significantly (*p* < 0.001) decreased after forced overexpression of miR-124 compared with the miRNA-negative control. Similarly, colony-formation assay demonstrated that the gain in function of miR-124 reduced the ability of transfected cells to self-renew (Figure 5b). In addition, the wound healing assay confirmed the influence of miR-124 released by PHB-PEI NPs on PC3’s migratory capability (78%, *p* < 0.01) compared to non-transfected cells (Figure 5c). Moreover, the number of PC3 cells crossing the Matrigel decreased of about 75% compared with the control cells (Figure 5d).

Together, the evidence demonstrates that miR-124 NPs represent a promising delivery system to target lipid oxidation in prostate and other cancers intervening in tumor progression and inhibition of metastasis and relapse.

## 3. Materials and Methods

### 3.1. Materials

Bacterial poly(3-hydroxybutyrate) (PHB), coded T19, was supplied by Biomer (Schwalbach, Germany). The number and weight average molecular weight, Mn and Mw, as determined by gel permeation chromatography (GPC), were 193 and 223 kg mol^−1^, respectively. Branched polyethylenimine (PEI) with an Mw of 800 kg mol^-1^ (800 D-PEI, ratio of primary:secondary:tertiary amine groups 1:0.82:0.7), Coumarin-6 (C6), and Pluronic F-127 were obtained from Sigma-Aldrich (Milan, Italy). Microfiltered (0.22 µm) double distilled water (milliQ) was used throughout the studies. Fetal bovine serum (FBS), Roswell Park Memorial Institute (RPMI) 1640 medium, Dulbecco’s modified Eagle’s medium (DMEM), sodium pyruvate, L-glutamine, penicillin, and streptomycin were purchased from Hyclone (Milan, Italy). All other chemicals and solvents used were of the highest grade commercially available (Sigma-Aldrich).

### 3.2. Synthesis and Functionalization of PHB NPs

PHB NPs were synthesized by nanoprecipitation, as previously reported [61], with minor modifications. Varying amounts of PHB were dissolved in N,N-Dimethylformamide (DMF) and the resulting solution was dropped in an aqueous solution of Pluronic F-127 under moderate magnetic stirring (solvent/non solvent ratio 1:5) (Table S1). The resulting suspension was kept under 300 rpm stirring overnight at room temperature, then centrifuged (18,000 rpm, 4 °C, 45 min). The obtained pellet was washed with milliQ water and, finally, freeze-dried. C6-loaded PHB NPs were prepared by adding C6 to the organic phase in a 10:1 polymer/drug ratio. Cationic PHB NPs were prepared through aminolysis to obtain PEI surface functionalized PHB nanoparticles (PHB-PEI NPs). Aminolysis was carried out by suspending PHB NPs in 800 D-PEI isopropanol solutions for a determined period of time at 50 °C. The effect of PEI concentration (from 12 to 48 wt %) and reaction time (0 to 60 min) on the amount of grafted PEI was systematically investigated. PHB-PEI NPs were recovered by centrifugation (13,300 rpm, 25 °C, 45 min), rinsed twice with distilled water, and freeze-dried.

### 3.3. Nanoparticles Characterization

Particle size and zeta potential were measured using a Zetasizer Nano ZS instrument (Malvern Instruments Ltd, Malvern, UK) on NPs dispersions in milliQ water. Size estimation with DLS was performed as follows: sampling time 60 s, temperature 25 °C, analysis with backscatter angle at 173°, viscosity of the dispersant 10,141 cp, and refraction index 1.5290. Zeta potential quantification with laser doppler micro-electrophoresis had the same settings. Smoluchowski approximation was used for the algorithm. Results are shown as mean ± SD (*n* = 3). Scanning electron microscopy (SEM) characterization of neat and PEI-functionalized PHB NPs were made with an FEI Quanta 200 FEG scanning electron microscope (Eindhoven, Netherlands). Prior to observation, the samples were transferred on an aluminum stub and coated with Au–Pd alloy by means of a sputtering device (MED 020, Bal-Tec AG) in order to provide a homogeneous layer of metal of 18 ± 0.2 nm. Transmission electron microscopy (TEM) analysis was carried out by an FEI Tecnai G2 Spirit TWIN 120 kV with emission source LaB6 and mounting FEI Eagle 4k CCD camera (on the bottom) and Olympus SIS MegaView G2 CCD Camera (on the side). NPs samples were prepared by casting a drop of nanoparticle water dispersion on a copper grid coated with a thin layer of carbon. Fourier transform infrared-attenuated total reflectance (FTIR-ATR) spectra were recorded on a Perkin Elmer Spectrum 100 spectrometer (Milan, Italy), equipped with a universal ATR diamond crystal sampling accessory. The spectra of PEI, as well as of PHB and PHB-PEI NPs, were acquired as an average of 64 scans in the range 4000–480 cm^−1^, with a resolution of 4 cm^−1^. Prior to measurements, the samples were kept at 60 °C under vacuum for 24 h. The amount of primary amino groups formed on the PHB-PEI NPs was determined by ninhydrin assay as described by Zhu et al. [38]. The NPs were solubilized in a 0.5 mL of CHCl3/DMF 3:1 *v/v* solution and mixed with 0.5 mL of 0.01 M ninhydrin solution (CHCl_3_/DMF 3:1 *v/v*). To promote the reaction between ninhydrin and the amino groups of the NPs, the solution was heated at 80 °C for 15 min. Once reacted, the sample turned blue. The absorbance of the solution was recorded at 558 nm using a Varian DMS 200 UV/Vis spectrophotometer (Milan, Italy). Standard solutions of 800 D-PEI (0.0001, 0.0005, 0.001, 0.005, 0.01, and 0.05 mM) were prepared and reacted with ninhydrin in CHCl_3_/DMF 3:1 *v/v* to obtain the calibration curve of standard solution. The absorbance of the PHB-PEI NPs dispersions was then compared with that of standard solution to determine the amine content of the PHB-PEI NPs.

### 3.4. PHB-PEI NPs Cytocompatibility

To assess PHB-PEI NPs cytocompatibility, a Cell Counting Kit-8 (CCK-8) assay was carried out. Briefly, PC3, Caco-2, MCF-7, and MCF10A cells were seeded in a 96-well plate in 100 μL at a density of 4 × 10^3^ cells/well 24 h before treatment. After 24 h, the cells were treated with 10 μL of PHB-PEI NPs at different concentrations (25–250 μg/mL) and incubated at 37 °C for 24–72 h. Then, 10 μL of CCK-8 solution were added to each well, and the plate was then incubated under cell culture conditions for 1–4 h. The optical density of formazan salt at 450 nm was measured using a Cytation 3 Cell Imaging Multi-Mode microplate reader (Biotek, Milan, Italy)). PHB-PEI NPs cytocompatibility was expressed as a percentage relative to the control and calculated by the equation:

Cytocompatibility (%) = (OD sample/OD control) × 100(1)

where OD sample is the optical density of cells treated with PHB-PEI NPs and OD control is the optical density of untreated cells.

### 3.5. PHB-PEI NPs/miRNA Complex (miR-124 NPs) Formation and Characterization

miR-124 NPs were investigated at various N/P ratios (ratio of amine groups of NPs to phosphate groups of miRNAs) from 0.5 to 20. Complexation between miRNAs (diluted in RNase-free DEPC-treated water) and NPs suspensions was achieved by gentle pipetting and incubation at room temperature for 30 min. To confirm complexation, RNase protection, and release ability, miR-124 NPs were loaded onto 2% agarose gels containing GelStar nucleic acid gel stain (Lonza, Milan, Italy) and gel electrophoresis assays were performed at 80 V for 40 min in Tris-acetate EDTA (TAE) buffer. Images were acquired using a Bio-Rad VersaDoc MP 4000 Molecular Imager (Milan, Italy). Quantity One 1.1 software (Bio-Rad) was used for band integration and background correction. The zeta potential and size of miR-124 NPs were measured by the Malvern Zetasizer Nano ZS equipment as described above. In vitro miRNA stability in culture medium and serum was performed as reported by Devulapally et al. [47] with some modifications. Briefly, 50 pmols of miRNA equivalent of miR-124-loaded NPs were dispersed in Dulbecco’s Modified Eagle’s Medium (DMEM) supplemented with 2% fetal bovine serum (FBS) and 1% penicillin and streptomycin and incubated at 37 °C and 5% CO_2_ for 0 to 1 days in a humidified atmosphere. At each time point, the reactions were terminated by adding 0.5 M of ethylenediaminetetraacetic acid and the samples were incubated at 37 °C for 1h in presence of 1% heparin sodium solution. Released free miRNAs from NPs were carefully separated by an ultracentrifuge filter device with 100 kDa MWCO Membrane (Millipore, Milan Italy) by centrifuging at 3000 rpm. The concentrated samples were washed and centrifuged several times with DNAse/RNAase free water (Invitrogen, Milan, Italy) and finally used for q-PCR, as reported in Section 3.7 and Section 3.8. For the RNase A protection assay, miR-124 NPs were incubated with 1 ng RNase A at 37 °C for 1 h. The nuclease activity of RNase A was terminated by treatment with 25 mM sodium dodecyl sulfate (SDS, Sigma-Aldrich) at 60 °C for 5 min. The absorbance of RNA at 260 nm was measured continuously for 1 h and the values were plotted vs. time.

### 3.6. Cell Culture

The human prostate adenocarcinoma cell line, PC3, as well as human adenocarcinoma cells (Caco-2), human breast carcinoma cells (MCF-7), and normal breast cells (MCF10A) were obtained from American Type Culture Collection (ATCC). PC3 and MCF10A were maintained in DMEM-F12, while the other cell lines were maintained in DMEM, all supplemented with 10% FBS, 1% l-glutamine, 50 U/mL penicillin, and 50 mg/mL streptomycin at 37 °C in a humidified atmosphere containing 5% CO_2_. MCF-10A were also supplemented with epidermal growth factor 20 ng/mL, cholera toxin 100 ng/mL, and insulin 10 µg/mL. Cells were tested for contamination, including mycoplasma, and used within 2 to 4 months.

### 3.7. miRNAs Transient Transfection

The day before transfection, 1×10^5^ cells were seeded under standard conditions into a 6-well plate in growth medium without antibiotics and transfected for 24 h using PHB-PEI NPs. Hsa-miR-124-3p, mirVana miRNA mimic (Ambion), and mirVana miRNA mimic negative control #1 (miR mimic NC, Ambion) were purchased from Applied Biosystems (Milan, Italy). For convenience, hsa-miR-124-3p mimic and the negative control are hereafter referred to as miR-124 and miR-NC, respectively. Forced expression of mimics were confirmed by qRT-PCR using TaqMan Universal PCR Master Mix (Applied Biosystem) and normalized to RNU6B. Transfected cells were used in further analyses.

### 3.8. RNA Isolation, Reverse Transcription, and Quantitative Real-Time PCR

Total RNA (mRNA and miRNAs) was extracted from cells after miR-124 NPs and miR-NC NPs transfection using QIAzol reagent (Qiagen, Milan, Italy) according to the manufacturer’s instructions. For retro-transcription, total RNA (0.2 μg) was treated as described in Promega standard protocol and amplified by qPCR using specific primers for carnitine palmitoyltransferase 1A (CPT1A) and β-actin (ACTB), as listed in Table 1. Quantitative PCR (qPCR) was run on a 7900HT fast real-time PCR System (Applied Biosystem). The reactions were performed according to the manufacturer’s instructions using SYBR Green PCR Master mix (Invitrogen). All reactions were run in triplicate, normalized to the housekeeping gene (ACTB), and the results expressed as mean ±SD. The 2^−∆∆*C*t^ method was used to determine the relative quantification.

### 3.9. Western Blotting

PC3 cells were grown to 70–80% confluence and, after miR-124 NPs and miR-NC NPs transfection, pellets were lysed in ice-cold buffer containing 50 mM Tris-HCl (pH 7.5), 125 mM NaCl, 1% NP-40, 5.3 mM NaF, 1.5 mM NaP, 1 mM orthovanadate, 175 mg/mL octylglucopyranoside, 1 mg·mL^−1^ protease inhibitor cocktail, and 0.25 mg·mL^−1^ 4-(2-aminoethyl) benzenesulfonyl fluoride hydrochloride (AEBSF). Cell pellets were centrifuged and the supernatants were resuspended in SDS sample buffer, then were normalized for equal protein concentration before separation by sodium dodecyl sulfate (SDS) polyacrylamide gel electrophoresis. SDS polyacrylamide gel electrophoresis and immunoblotting were carried out according to standard procedures in triplicate, using 30 μg of total protein lysate. Antibodies were mouse monoclonal anti–carnitine palmitoyltransferase 1A (1:1000, Abcam, Milan Italy) and mouse monoclonal Anti-β-Actin (ACTB, 1:1000, Sigma-Aldrich). Anti-mouse was used as a secondary antibody (1:5.000, Santa Cruz, Milan Italy). The relative expression, normalized with respect to the housekeeping protein (ACTB), was quantified densitometrically using Quantity One 1-D analysis software (Bio-Rad).

### 3.10. miR-124 NPs Cell Viability and Colony Forming Assay

To assess miR-124 NPs-transfected cell proliferation, a CCK-8 assay was performed. PC3 cells were grown in 96-well plates at a density of 2.0 × 104 cells/well. Cells were treated with miR-124 NPs suspended in complete culture medium and diluted to the appropriate concentration (25–250 µg/mL). Cell proliferation at 0, 24, 48, and 72 h was determined by CCK8 assay following the manufacturer’s protocol. The absorbance of each well was measured with a microplate reader (Cytation3, ASHI) at 450 nm. For colony formation assay, cells were counted and seeded in 6-well plates (in triplicate) at a density of 500 cells/well. Cells were treated with PHB-PEI NPs and miR-124 NPs at 50 µg/mL. The plates were incubated at 37 °C in a 5% CO_2_ humidified incubator. The culture medium was replaced every 3 days. After 14 days in culture, cells were stained with crystal violet and counted. Colonies with at least 50 cells were considered for quantification. Representative plates were photographed using a phase contrast microscope (Leica, Milan, Italy).

### 3.11. Cellular Uptake of miRNA

To assess the cellular uptake and binding of miR-124, PC3 cells were seeded on a microscope slide at a density of 4.5 × 104 cells in 24-well plates. Then, they were incubated with 100 nM of free Cy5-miRNA or PHB-PEI/Cy5-miRNA nanocomplexes at 37 °C for 0, 15, and 60 min. Cells were then washed 2 times with phosphate-buffered saline (PBS) and incubated in fixing solution (3.7% formaldehyde in PBS) for 15 min at room temperature. The microscope slides were mounted onto coverslips using ProLong Gold Antifade reagent with DAPI (Thermo Fisher Scientific, Milan, Italy). The percentage of miRNA uptake and binding was determined by measuring FAM-miRNA positive cells. NPs uptake was visualized by a Cytation 3 Cell Imaging Multi-Mode Reader fluorescent microscope (Biotek) at 520 nm.

### 3.12. miR-124 NPs Tumor Cell Migration and Invasion Assays

A scratch wound healing model was conducted to check the migratory ability of PC3 cells following the transfection treatment. PC3 cells were plated at a density of 1 × 105 cells in 2-well Lab-Tek Chamber Slide (Sigma-Aldrich). After overnight incubation, cells were transfected with mimics or miR-NC. Wounds were created in confluent cells using a 200 μL pipette tip. The cells were rinsed several times with media to remove any free-floating cells or debris and the speed of wound closure was monitored after 24 h by measuring the change in distance of the wound edges. Each experiment was conducted in triplicate and representative scrape lines were photographed using a phase contrast microscope (Leica). For the invasion assays, after 24 h transfection, 1 × 105 cells in serum-free media were seeded onto the transwell migration chambers (8 μm pore size, Millipore). The insert in the upper chamber was coated overnight with 1 mg·mL^−1^ BD Matrigel Matrix (BD Biosciences, Milan, Italy). A medium containing 10% FBS was added to the lower chamber. After 24 h, the non-invading cells were removed with a cotton-tipped swab and cells at the bottom of the Matrigel were stained with May–Grunewald–Giemsa stain (Sigma-Aldrich). Stained cells were counted under a microscope (Leica) at 200X magnification in 5 random fields in each well. Experiments were independently repeated three times.

### 3.13. Statistical Analyses

All values are expressed as mean ± standard deviation (SD). Each experiment was performed at least three times. A one-way analysis of variance (ANOVA) was used for statistical analysis, followed by Bonferroni’s test for multiple comparisons to determine significance differences between groups. All the data were analyzed with the GraphPad Prism version 6.01 statistical software package (San Diego, CA, USA)

## 4. Conclusions

Aminolysis is a straightforward and easy-to-perform route to prepare PHB-based cationic NPs. The latter are successfully used as non-viral vectors to deliver miR-124 in androgen-independent prostate cancer (PC3) cells. The functionalization of PHB NPs with low-molecular-weight PEI results in NPs with high miR-124 loading capacity, which exhibit no significant cytotoxicity effect, even after 72 h. Furthermore, optimization of the N/P ratio contributes to a stable and uniform miR-124 NPs dispersion in aqueous solution and effectively protects miR-124 from RNAse degradation. PHB-PEI NPs showed 30% higher transfection efficiency compared to Lipofectamine RNAiMAX. A dramatic impairment of miR-124 NPs-transfected PC3 cell proliferation, motility, and colony formation was found due to the downregulation of CPT1A at both the transcriptional and translation level compared to non-transfected cells. Furthermore, the tumor-targeting efficacy of the reported delivery system should be assessed in in vivo models to validate the use of PHB-PEI NPs in non-invasive treatment of prostate cancer.

## Figures and Tables

**Table 1:** Sequence of primers used in real-time polymerase chain reaction.

Genes	Forward primers (5′–3′)	Reverse primers (5′–3′)
*ACTB*	TTAGTTGCGTTACACCCTTTC	ACCTTCACCGTTCCAGTT
*CPT1A*	CTGGACAATACCTCGGAGCC	TCTAACGTCACGAAGAACGCT

## Supplementary Materials

Supplementary Materials can be found at https://www.mdpi.com/1422-0067/21/3/869/s1.
