# Supplementary material for: Cationic Polymer Nanoparticles-Mediated Delivery of miR-124 Impairs Tumorigenicity of Prostate Cancer Cells
Source: Int J Mol Sci. 2020 Jan 29;21(3):869. doi: 10.3390/ijms21030869 (PMC7038067; doi:10.3390/ijms21030869)
Supplement: Supplementary file 1 [file ijms-21-00869-s001.pdf]

Supporting Information

Cationic polymer nanoparticles mediated delivery of miR-124 impairs tumorigenicity of prostate cancer cells

Raffaele Conte,[a,‡] *Anna Valentino,*[b,‡] *Ilenia De Luca,*[b] *Gianfranco Peluso,*[a] *Pierfrancesco Cerruti,*[c,*] *Anna Di Salle,*[a] *Anna Calarco*[a,*]

[a]Research Institute on Terrestrial Ecosystems (IRET-CNR), Via P. Castellino 111 80131 Napoli, Italy

[b]Elleva Pharma Srl, Via P. Castellino 111 80131 Napoli, Italy

[c]Institute for Polymers, Composites and Biomaterials (IPCB-CNR), Via Campi Flegrei 34 80078 Pozzuoli (Na), Italy

Correspondence to: Pierfrancesco Cerruti. email: cerruti@ipcb.cnr.it; Anna Calarco. email: anna.calarco@cnr.it

Contents

Table S1. Size of PHB NPs (nm) as a function of PHB and Pluronic® F-127 concentration..S3

Table S2. Concentration (mmol $-NH_2$/g) of $-NH_2$ groups on the PHB-PEI NPs surface....................S4

Figure S1. Evaluation of cytotoxicity in Caco-2, MCF-7 and MCF10A cells after 6, 24, 48 and 72 hours of incubation with PHB-PEI NPs ……………………………………………………….…….S5

Figure S2. Degradation assay of free miRNA or miR-124 NPs at N/P ratio of 1:1, 5:1, and 10:1 treated with RNase A for 60 minutes. Released nucleotides was measured by spectrophotometric continuous detection (λ = 260 nm)…………………………………………………………………S6

Table S1. Size of PHB NPs (nm) as a function of PHB and Pluronic® F-127 concentration.

PHB concentration (mg/ml)	Pluronic® F-127 concentration (wt%)			
	0.1	0.5	1	2
8.33	298.9 ± 48.8	153.3 ± 44.1	191.7 ± 49.7	138.3 ± 44.9
16,7	455.4 ± 95.7	254.5 ± 43.2	231.4 ± 55.0	167.7 ± 44.1
33,3	644.6 ±115.8	425.2 ± 87.9	381.0± 65.0	305.4 ± 59.1
41.7	705.5 ± 235.8	554.4 ± 97.7	502.5± 85.4	450.3 ± 73.5

Table S2. Concentration (mmol $-NH_2$/g) of $-NH_2$ groups on the PHB-PEI NPs surface.

PEI concentration (% w/v)	Reaction conditions											
	5 min at 25 °C	15 min at 25 °C	30 min at 25 °C	60 min at 25 °C	5 min at 50 °C	15 min at 50 °C	30 min at 50 °C	60 min at 50 °C	5 min at 100 °C	15 min at 100 °C	30 min at 100 °C	60 min at 100 °C
12	12.3	15.4	14.4	13.4	33.1	32.2	34.3	36.4	29.3	30.3	30.4	31.2
24	13.3	16.4	14.4	15.2	31.3	38.2	36.4	35.3	34.2	34.1	32.4	33.3
48	11.2	14.1	11.4	10.2	35.1	33.2	34.3	33.3	33.4	31.2	29.1	26.3

Figure S1

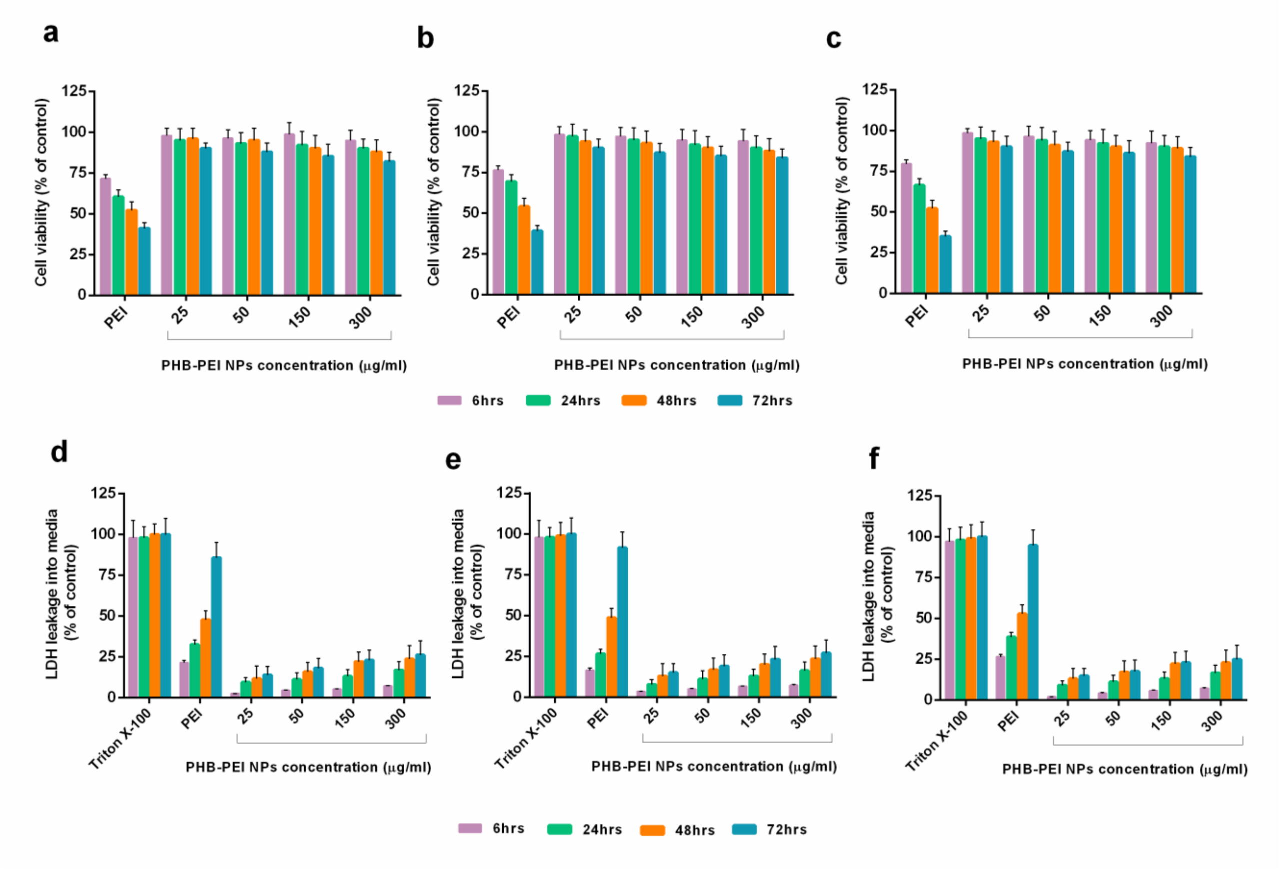

Figure S1. Cytotoxicity was determined in Caco-2, MCF-7 and MCF10A cells after 6, 24, 48 and 72 hours of incubation with varying concentrations of PHB-PEI NPs using the CCK-8 assay: (a, b and c; Caco-2, MCF-7 and MCF10A respectively), and the LDH assay (d, e and f; Caco-2, MCF-7 and MCF10A respectively). Untreated cells were used as control. Data represent the mean±SD for three independent measurements.

Figure S2

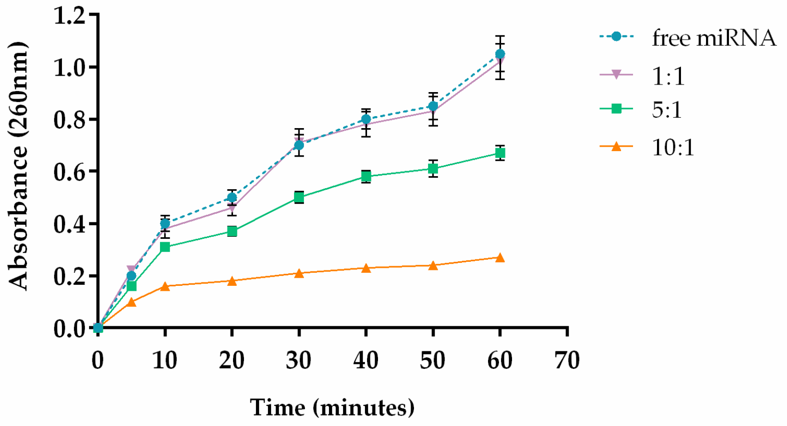

Figure S2. Degradation assay of free miRNA or miR-124 NPs at N/P ratio of 1:1, 5:1, and 10:1 treated with RNase A for 60 minutes. Released nucleotides were measured by UV spectrophotometric continuous detection (λ = 260 nm).
